# Draft Genome Sequence of the Cellulolytic, Mesophilic, Anaerobic Bacterium *Clostridium termitidis* Strain CT1112 (DSM 5398)

**DOI:** 10.1128/genomeA.00281-13

**Published:** 2013-05-23

**Authors:** Sadhana Lal, Umesh Ramachandran, Xiangli Zhang, Riffat Munir, Richard Sparling, David B. Levin

**Affiliations:** Department of Biosystems Engineering, University of Manitoba, Winnipeg, Manitoba, Canadaa; Department of Microbiology, University of Manitoba, Winnipeg, Manitoba, Canadab; Department of Plant Science, University of Manitoba, Winnipeg, Manitoba, Canadac

## Abstract

Here, we report the draft genome sequence of *Clostridium termitidis* strain CT1112 (DSM 5398), a mesophilic, cellulolytic bacterium that can utilize a variety of sugars, as well as pure cellulose, as a sole carbon source; it also synthesizes fermentation end products with potential industrial applications.

## GENOME ANNOUNCEMENT

*Clostridium termitidis* strain CT1112 (DSM 5398) is a Gram-positive, mesophilic, anaerobic, cellulolytic bacterium isolated from the gut of the wood-feeding termite *Nasutitermes lujae* (1). Based on its 16S rRNA, *C. termitidis* belongs to *Clostridium* cluster III (2). It can utilize a wide variety of substrates, such as cellulose, cellobiose, glucose, fructose, and many other sugar monomers, as a sole carbon source, and it produces hydrogen (H_2_), carbon dioxide (CO_2_), acetate, formate, lactate, and ethanol as major fermentation end products (1, 3).

The genome of *C. termitidis* CT1112 was sequenced by the Genome Québec/McGill University platform using a Roche/454s GS-FLX Titanium sequencer by a whole-genome shotgun strategy, which obtained 303,437 reads. A 454 standard flowgram format (.sff) read file was assembled using Newbler v2.3. The final draft genome assembly has approximately 17-fold coverage and contains 78 contigs (>800 bp in length), with a total size of 6,415,858 bp, an *N*_50_ contig length of 146,289 nucleotides, and a mean G+C content of 41.18%. The draft genome sequence was automatically annotated using IMG-ER, an online system developed by the U.S. Department of Energy Joint Genome Institute (JGI) (http://www.jgi.doe.gov/). The IMG-ER annotation was processed by a JGI-developed Gene Prediction Improvement Pipeline (GenePRIMP) (4) and was further subjected to manual curation using Artemis (5). The draft genome sequence of *C. termitidis* is estimated to have a total of 5,389 genes, including 5,302 protein-coding genes, 55 tRNAs, and 7 rRNAs.

The *C. termitidis* genome is larger than those of other mesophilic and thermophilic cellulolytic *Clostridium* spp., such as *Clostridium cellulolyticum* H10 (4,068,724 bp), *Clostridium cellulovorans* 743B (5,262,222 bp), *Clostridium phytofermentans* ISDg (4,847,594 bp), *Clostridium thermocellum* ATCC 27405 (3,843,301 bp), and *C. thermocellum* DSM 1313 (3,561,619 bp). The G+C content of *C. termitidis* is higher (41.18%) than those of other cellulolytic *Clostridium* species (31.21% to 39.15%), as is the number of predicted genes (5,389).

*C. termitidis* protein-coding genes were verified using other cellulolytic *Clostridium* species as reference organisms. Amino acid sequences for each gene product were retrieved from the JGI genome portal (http://genome.jgi-psf.org/) (6) and the NCBI database (http://www.ncbi.nlm.nih.gov/genomes/lproks.cgi), and sequence alignments against *C. termitidis* genes were performed. The corresponding gene loci and enzymes for each pathway were identified by percent amino acid sequence identity and were based on a conserved domain of proteins (7). In this manner, the key enzymes involved in *C. termitidis* core metabolism, as well as the major cellulosomal components and glycoside hydrolases, were identified. *C. termitidis* has potential as an industrial microorganism for the production of biofuels and/or other value-added products through direct cellulose fermentation via consolidated bioprocessing.

### Nucleotide sequence accession numbers.

The genome sequence of *C. termitidis* strain CT1112 (DSM 5398) has been deposited at DDBJ/EMBL/GenBank under the accession no. AORV00000000. The version described in this paper is the first version, accession no. AORV01000000.
